# New Appliances and Things Medical

**Published:** 1907-03-30

**Authors:** 


### NEW APPLIANCES AND THINGS MEDICAL.

[We shall be glad to receive at our Office, 28 & 29 Southampton Street, Strand, London, W.C., from the manufacturers, specimens of all new preparations and appliances which may be brought out from time to time.]

### INJECTIO TRYPSINI AND INJECTIO AMYLOPSINI.

(FAIRCHILD BROTHERS AND FOSTER, NEW YORK. LONDON AGENTS, BURROUGHS WELLCOME AND COMPANY.

MESSRS. FAIRCHILD BROTHERS AND FOSTER have kindly sent us samples of the above injections, each packet containing twelve ampoules. The contents of the ampoule are liberated in the usual manner—namely, by fracturing its glass neck. Each ampoule contains approximately 20 minims, and for the purpose of the injection should be diluted with twice its volume of sterilised distilled water, this procedure rendering the fluid to be injected isotonic with normal saline solution. These elegant preparations are specially designed for use in the trypsine treatment of cancer. Our readers will no doubt be acquainted with the literature of this subject and with the record of those cases of cancer which have apparently shown distinct improvement during and after a course of trypsine. From the experience at present available it appears that at a certain stage of the treatment injections of "amylopsin" should be substituted for those of trypsine. With the object of supplying a standard preparation of the latter ferment—*i.e.* pancreatic diastase—Messrs. Fairchild Brothers and Foster have put on the market their ampoules of injectio amylopsini, this latter being absolutely free from both the proteolytic and the fat-splitting ferments of the pancreas. The usual dose of these injections is from 5 to 20 minims, at first twice a week, then every other day, and finally once or even twice a day.

In our opinion any medical man wishing to try this treatment cannot do better than make use of the products which we have been discussing, as they are certainly most carefully and accurately made, and should yield the optimal therapeutic results.

